# Experimental and Numerical Study of the Ultimate Flexural Capacity of a Full-Size Damaged Prestressed Concrete Box Girder Strengthened with Bonded Steel Plates

**DOI:** 10.3390/ma16062476

**Published:** 2023-03-20

**Authors:** Yong Li, Zijie Yu, Yongqian Liu

**Affiliations:** The Key Laboratory of Roads and Railway Engineering Safety Control, School of Civil Engineering, Shijiazhuang Tiedao University, Shijiazhuang 050043, China

**Keywords:** prestressed concrete box girder, concrete damage, steel bars and strand fracture, ultimate flexural bearing capacity, destructive test, repair and strengthening

## Abstract

Using steel plates attached with epoxy resin adhesive to strengthen prestressed reinforced concrete bridges has become a common method to increase bearing capacity in engineering because of the simple technology, low cost and good strengthening effects. The strengthening method of steel plates has been gradually applied to repair damaged bridges in practical engineering. After a cross-line box girder bridge was struck by a vehicle, the steel bars and concrete of a damaged girder were repaired and strengthened by steel plates, and then the ultimate bending bearing capacity was studied through a destructive test. The results of the destructive test were compared with those of an undamaged girder to verify the effect of the repair and strengthening of the damaged girder. The results showed that the actual flexural bearing capacity of the repaired girder strengthened by steel plates was 1.63 times the theoretical bearing capacity, 36.7% more than that of the damaged girder and 95.3% of that of an undamaged girder. The flexural cracking moment of the repaired girder strengthened by steel plates reached 66.3% of that of the undamaged girder. The maximum crack width decreased by 24.6%, and the maximum deflection increased by 2.7%, compared with the undamaged girder when the repaired girder strengthened by steel plates finally failed. Moreover, this method of attaching steel plates can increase the ductility of bridges and reduce the degree of cracking. Additionally, the actual safety factor of the repaired girder was greater than three, and it had a large safety reserve.

## 1. Introduction

With the accelerated pace of urban construction and the continuous rise in the number of cars, the cross-line bridge has become an essential structure to improve city traffic, relieve the pressure of urban traffic and improve the efficiency of road transport [1]. However, cross-line bridges are often struck by vehicles to varying degrees [2], which will cause cracks in the girder body and even damage to the steel bars, concrete and post-tensioned strands [3,4,5]; as a result, their flexural capacity does not meet the design load specified in the current design specification [6]. To reduce the effects of traffic on in-service bridges after they undergo collisions, and prevent large areas of traffic paralysis, there is a need for new methods to repair and strengthen or demolish and reconstruct bridges [7]. Demolition and reconstruction require very large financial input and cause severe environmental pollution. Compared with demolition and reconstruction, repairing and strengthening in-service bridges have the advantages of economy, environmental protection and less impact on road traffic. Therefore, repairing and strengthening in-service bridges have become common methods to improve the bearing capacity and restore damaged in-service bridges [8,9].

At present, reinforcement by steel plates is widely used because of its advantages, such as fast construction, low cost and remarkable strengthening effect, for prestressed bridges [10]. The steel plates are generally bonded to the girder by a structural adhesive or epoxy resin, and anchored on the tensile edge or the weak surface of the bridge to form a common force with the bridge as a whole, and then play the role of steel bars which increase the strength [11]. Compared with strengthening by bonding fibers, strengthening by bonding steel plates can make full use of the mechanical properties of steel plates [12]. Steel plates are easy to obtain and relatively inexpensive [13], and they have the material characteristics of uniform stress and good plasticity [14]. Strengthening by steel plates has been proven to effectively improve the stiffness, reduce the deformation under live loads [15], enhance the crack resistance [16], and more importantly, effectively improve the bending [17] and shear performance of the main girder [18], and it has no significant impact on the appearance of the structure and headroom [19]. Most of the previous conclusions on the strengthening effect of bonded steel plates were obtained for undamaged girders, but there were few relevant studies on the damaged and cracked prestressed concrete box girders that have been repaired [20,21]. Even if there are, the bearing capacity is analyzed through refined finite element analysis, and no full-bridge destructive test is carried out [22,23]. Therefore, this paper evaluates the bearing capacity of the damaged girder after repair through the full-bridge destructive test.

We take a prestressed concrete box girder bridge as the research object, specifically the third and fourth spans of the cross-line that were severely damaged when struck by a truck. The mechanical properties of the damaged and undamaged fourth span box girders were compared previously [24]. The technical status of the bridge was assessed, and the bearing capacity of the third span in the damaged girder was checked according to the specification [6]. The decision was made to use steel with the same strength as the steel bars to repair the damaged main steel bars, and stirrup and concrete with the same strength as the original concrete was used to repair the concrete of the bottom plate, and at the same time, the cracks were closed and strengthened with steel plates [25]. To study the ultimate bearing capacity of the damaged girder after repair and strengthening, destructive tests were conducted on an undamaged girder and the repaired girder strengthened by steel plates [26,27,28,29,30], and the destructive process was simulated through detailed analysis [31,32]. The actual bearing capacity of the repaired girder strengthened by steel plates was evaluated by contrasting the data of the destructive tests and a thorough study of the two girders. The results can serve as a guide for future evaluations of the bearing capacity of similarly damaged bridges.

## 2. Engineering Situations

### 2.1. Bridge Information

**Figure 1:**
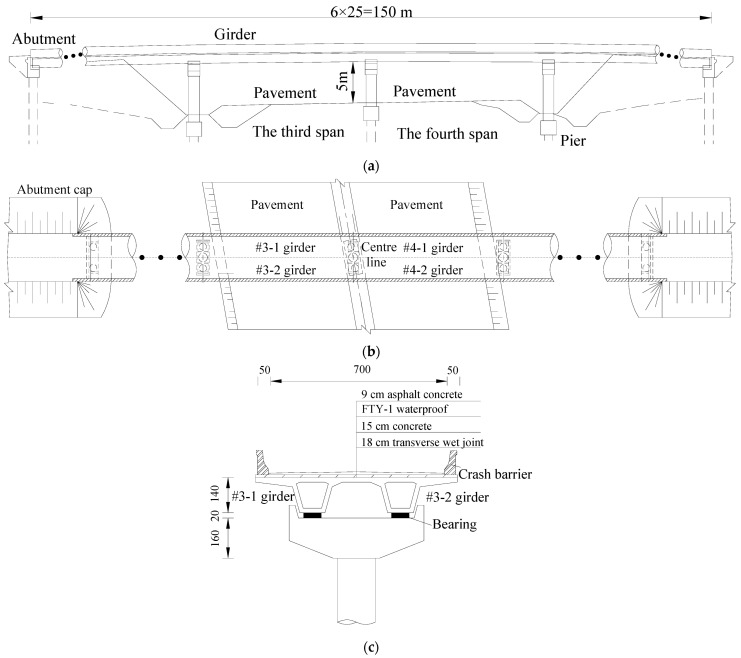
Details of the entire bridge arrangement. (**a**) Elevation arrangement (Unit: m); (**b**) Plane layout; (**c**) Cross-sectional layout (Unit: cm).

**Figure 2:**
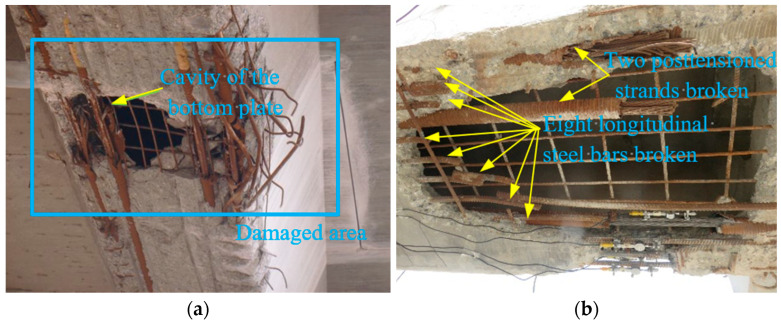

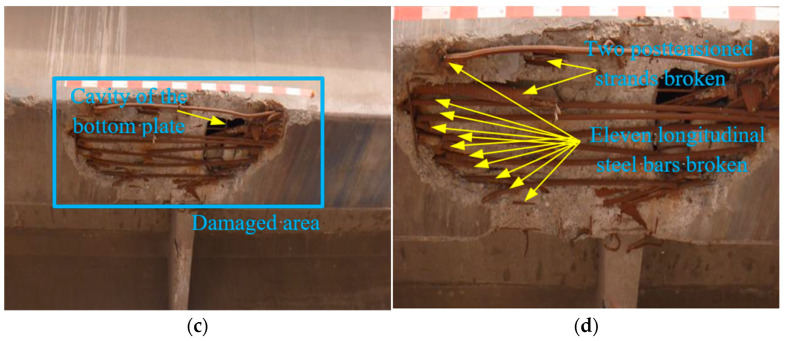
Modes of damage at the bottom of box girders. (**a**) Damaged area of the #4-2 girder; (**b**) Steel bars and pretensioned strands broken in the #4-2 girder; (**c**) Damaged area of the #3-2 girder (**d**) Steel bars and pretensioned strands broken in the #3-2 girder.

The basic information of the bridge is detailed in a reference [24], and Figure 1 shows the whole layout of the bridge in detail. The expressway is crossed by the third and fourth spans of the bridge, and the traffic flow under the bridge is large. Due to a collision by an overhigh vehicle, a girder was severely damaged in the third span and another in the fourth span, as shown in Figure 2. The damaged #4-2 girder and the undamaged #4-1 girder of the fourth span were compared and studied [24]. In this paper, the bearing capacity of the damaged #3-2 girder of the third span, which was repaired and strengthened with steel plates, was studied. The undamaged #3-1 girder of the third span, which was strengthened with carbon fiber reinforced plastic (CFRP) plates, will be researched in a future study.

### 2.2. Appearance of Damage

Due to the collision with the overhigh vehicle, the prestressed concrete box #4-2 and #3-2 girders were severely damaged. The significant modes of damage of the #4-2 girder are shown in Figure 2a,b [17], and the main modes of damage of the #3-2 girder are shown in Figure 2c,d. A total of 1.82 m^2^ of concrete fell off, and a cavity appeared in the bottom plate of the #4-2 girder. Additionally, 2 m^2^ of concrete were damaged 2~4.5 m from the middle span of the #3-2 girder, but a cavity of only 0.4 m^2^ formed. In the bottom plate of the #4-2 girder, eight longitudinal steel bars and two pretensioned strands broke. In addition to the fracture of these steel bars and pretensioned strands, three more longitudinal steel bars broke in the #3-2 girder than in the #4-2 girder. Moreover, the unbroken steel bars and pretensioned strands in the damaged areas of the two girders were exposed to air due to the loss of concrete.

**Figure 3:**
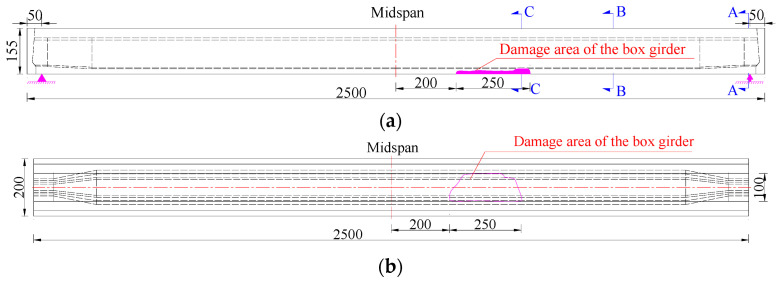

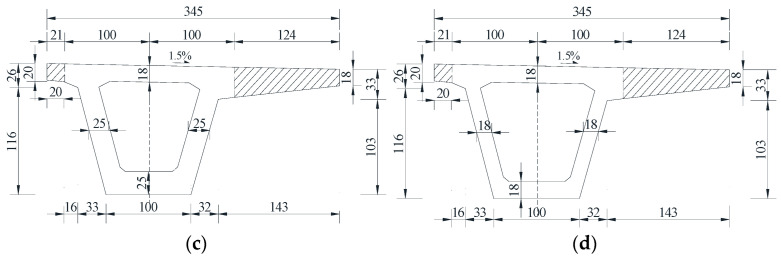
Cross-sectional diagrams of the box girders at the third and fourth spans (Unit: cm). (**a**) Front view; (**b**) Bottom view; (**c**) A-A section; (**d**) B-B section.

**Figure 4:**
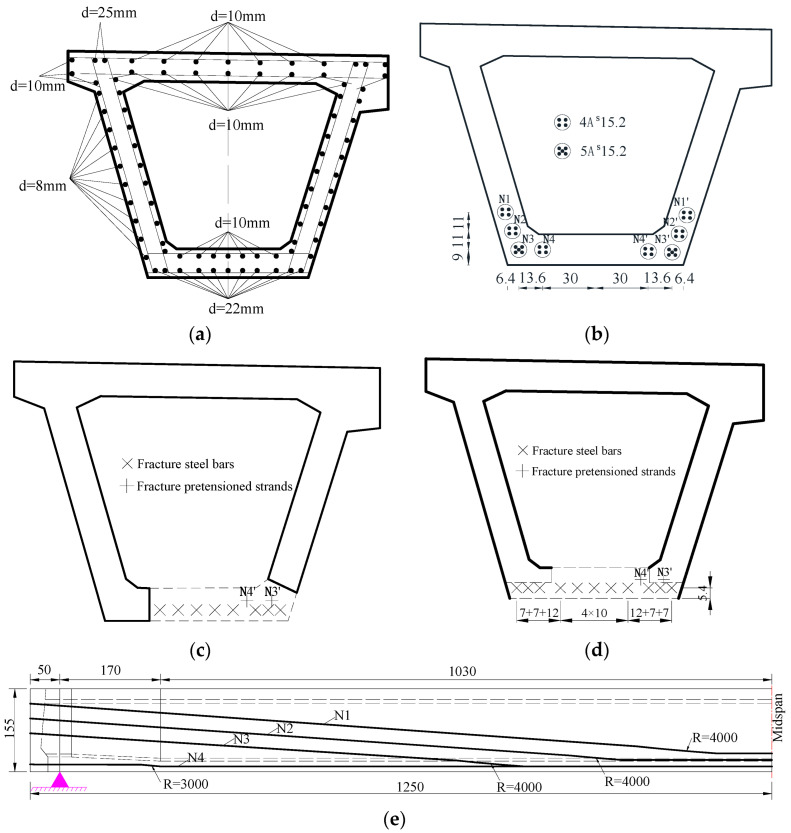
Arrangement of steel bars and strands in the girder (Unit: cm). (**a**) Steel bars in the undamaged girder (B-B); (**b**) Pretensioned strands in the undamaged girder (B-B); (**c**) Steel bars in the damaged region of the #4-2 girder (C-C); (**d**) Steel bars in the damaged region of the #3-2 girder (C-C); (**e**) Strands arrangement.

To facilitate the transportation of the prestressed concrete box girders and the destructive tests, and considering that the overstretched flange plates of box girders had little influence on bearing capacity, the flange plates on both sides of the box girders were partially removed. The cut girders were supported simply at the girder end, as indicated in Figure 3. Figure 4a,b show the arrangement of the steel bars and pretensioned strands of the B-B section in the box girder. Figure 4c,d show the steel bars and pretensioned strands that broke in the C-C section of the #4-2 and #3-2 girders, respectively, whereas Figure 4e shows the longitudinal arrangement of the pretensioned strands.

## 3. Theoretical Analysis

### 3.1. Flexural Capacity

**Figure 5:**
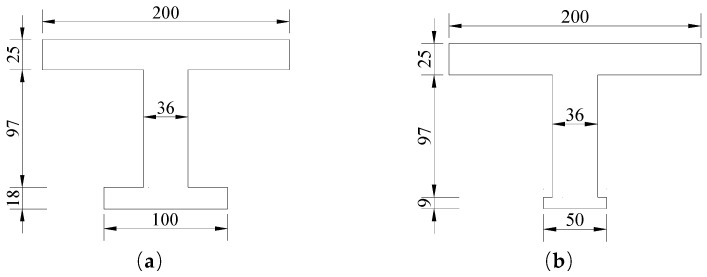
Equivalent sections of the #3-2 girder in undamaged, damaged and repaired states (Unit: cm). (**a**) Undamaged girder and repaired girder; (**b**) Damaged girder.

The bearing capacity of the #3-2 girder in undamaged, damaged and repaired states was calculated to determine whether it needed to be strengthened. First, the calculated section was determined before the ultimate bending capacity was calculated. Due to the adoption of a simply supported system at the girder end, the computed section of the undamaged #3-2 girder was selected as the midspan section. The calculated section was determined to be the section with the most severe damage for the damaged #3-2 girder and the repaired #3-2 girder since the damaged area was close to the midspan. In the repaired girder, the same strengths of steel bars and concrete were used to repair the broken longitudinal steel bars, stirrups and concrete of the bottom plate, and the cracks were closed. The box section was equivalent to an I-shape when the flexural bearing capacity was computed, according to the specification [6]. Figure 5a depicts the equivalent sections of the undamaged and repaired box girders, and Figure 5 shows the equivalent section of the damaged girder (b).

According to the design drawings, specifications and results of the appearance inspection, the calculated parameters of the #3-2 girder in undamaged, damaged and repaired states were obtained, as shown in Table 1. According to the specification [6], Equation (1) was satisfied by the calculated sections of the damaged, undamaged and repaired girders, and Equation (2) was employed to calculate the flexural bearing capacity Mu. The undamaged girder’s calculated section had a flexural bearing capacity Mu of 8442 kN/m and a cracking bending moment of 6984 kN/m. The flexural bearing capacities of the damaged and repaired girders were 5127 kN·m and 6432 kN·m, respectively, which were 39.3% and 23.8% less than that of the undamaged girder. However, the result for the repaired girder was 25.5% larger than that of the damaged girder.

(1) $$f_{sd}A_{s}+f_{pd}A_{p}\leq f_{cd}b_{f}^{\prime }h_{f}^{\prime }+f_{\mathrm{sd}}^{\prime }A_{s}^{\prime }$$

(2) $$M_{u}=f_{cd}b_{f}^{\prime }x(h_{0}-\frac{x}{2})+f_{sd}^{\prime }A_{s}^{\prime }(h_{0}-a_{s}^{\prime })$$

where *f*_sd_ and *f*^′^_sd_ are design tensile strength and compressive strength of longitudinal steel bars. *f*_pd_ is design tensile strength of strands. *f*_cd_ is design compressive strength of concrete. *A*_s_ and *A*^′^_s_ are areas of longitudinal reinforcement. *A*_p_ is section area stands. *h*^′^_f_ and *b*^′^_f_ are thickness and width of flanges for the equaled I-shape. *h*_0_ is the effective height. *x* is the height of the compressive zone. *a*^′^_s_ is the distance between the area centroid of compression reinforcement and concrete edge.

### 3.2. Load Effect under the Designed Load

**Figure 6:**
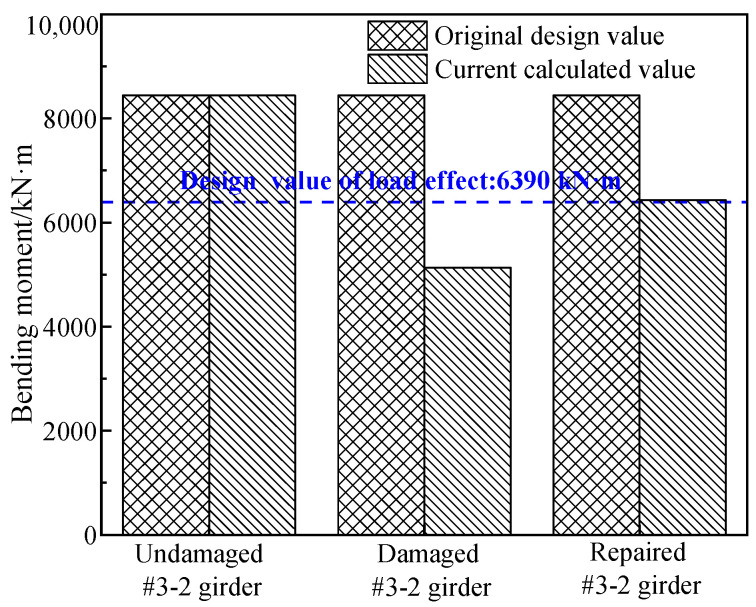
Comparison between the calculated flexural capacities and the design load effect value.

The value of the design load effect of the prestressed concrete box girder was calculated in order to figure out if the flexural bearing capacity of the damaged #3-2 girder and repaired #3-2 girder satisfied the requirements of the current bridge design specification [6]. According to the design drawings of the girder, the design load was determined to be road-II level, and the calculated bridge load effect combination was determined to be 1.2 times the dead load plus 1.4 times the road-II level, according to the specification. The effect of the prestressed concrete box girder’s design load was 6390 kN·m. The comparison between the theoretical values of the flexural bearing capacities of the three box girders and the design load effect value is shown in Figure 6.

Figure 6 shows that the theoretical value of the damaged #3-2 girder’s flexural bearing capacity was 19.8% less than the value of the design load effect of the original #3-2 girder, which indicated that the damaged #3-2 girder was a hidden danger and a great risk to safety, and the vehicle impact had a massive effect on the bridge’s bearing capacity. The bearing capacity of the repaired #3-2 girder was calculated to be 6432 kN·m, which was similar to the design load effect of the original #3-2 girder of 6390 kN·m. This indicated that the safety reserve of the repaired #3-2 girder was insufficient, which limited its capacity. It was necessary to strengthen the repaired #3-2 girder by steel plates.

### 3.3. Strengthening Scheme of the Repaired Girder

**Figure 7:**
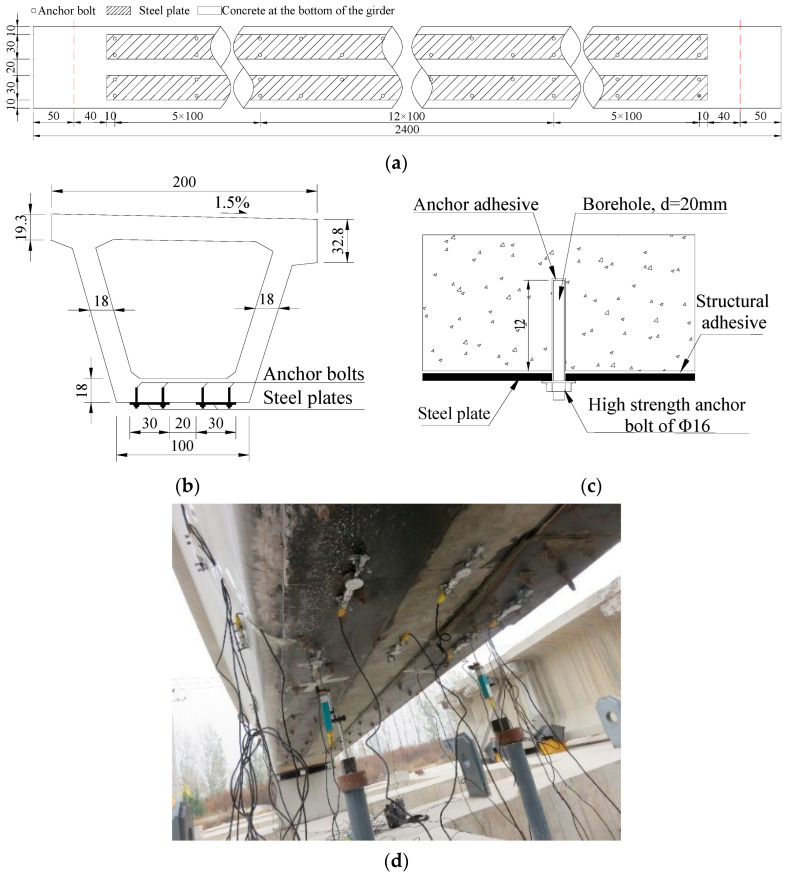
Layout of steel plates. (**a**) Layout at the bottom of the girder (Unit: cm); (**b**) Cross-sectional view of the steel plates layout (Unit: cm); (**c**) Details of the layout of steel plates; (**d**) Actual steel plates at the bottom of the girder.

**Figure 8:**
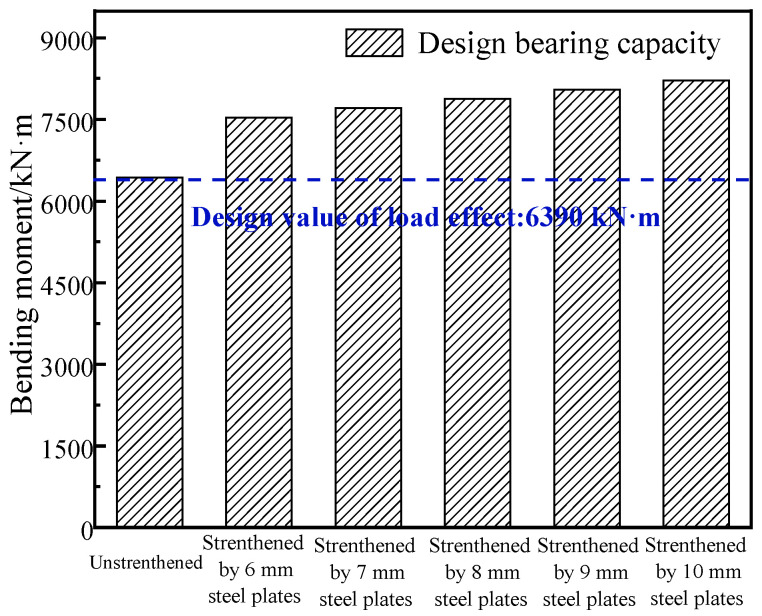
Comparison of the bearing capacities of the #3-2 girder strengthened by various steel plates.

Table 2 shows the performance parameters of steel plate Q345 that was selected for strengthening. Considering the width of the bottom of the prestressed concrete girder, two steel plates were selected for strengthening the girder. The width of a single steel plate was 30 cm, and the effective length was 2200 cm, as shown in Figure 7. According to the specification [33], the elastic modulus was 210 GPa, the design value of the axial tensile strength was 275 MPa and the measured tensile strength of the strengthening steel plates was 385 MPa. According to the strengthened specification [34], the strengthened effect of the thickness of the 6-10 mm thick steel plate on the repaired box girder was calculated using Equations (3) and (5), and Figure 8 shows the results. Considering the field test conditions and the results of the strength analysis, an 8 mm thick steel plate was finally selected to reinforce the repaired #3-2 girder.

(3) $$f_{cd}bx+{f^{\prime }}_{sd}{A^{\prime }}_{s}=f_{sd}A_{s}+f_{pd}A_{p}+\psi_{sp}f_{sp}A_{sp}$$

(4) $$2{a^{\prime }}_{s}\leq x\leq \xi_{b}h_{0}$$

(5) $${M^{\prime \prime \prime }}_{u}=f_{cd}bx\left(h_{0}-\frac{x}{2}\right)+{f^{\prime }}_{sd}{A^{\prime }}_{s}\left(h_{0}-{a^{\prime }}_{s}\right)+\psi_{sp}f_{sp}A_{sp}$$

where *f*_sp_ is the design tensile strength of the steel plates. *A*_sp_ is the area of the steel plates. *A*_p_ is the section area of the strands. *Ψ*_sp_ is the influence coefficient of the bearing capacity of the bonded steel plate when second-stage stress and cracks in the strengthened girder are considered; the value ranged from 0.85 to 0.95, according to the maximum width of the surface cracks before strengthening. Finally, *ξ*_b_ is the balanced relative depth of the compressive area.

Considering the strengthened analysis results and field test conditions, an 8 mm thick steel plate was finally selected to reinforce the repaired #3-2 girder. The main construction steps of attaching the steel plates were as follows: First, the bottom concrete of the girder was grinded and cleaned to make the surface smooth and flat. Then, the positions of two steel plates were determined at the bottom of the girder, and they were anchored with Q345 high-strength anchor. Glue was spread between the steel plates as well as the concrete using the pressure glue injection method to firmly bond the two surfaces. Finally, the steel plates were brushed with protective paint to prevent corrosion.

## 4. The Refined Finite Element Analysis

### 4.1. Establishment of the Model of the Undamaged #3-2 Girder

**Figure 9:**
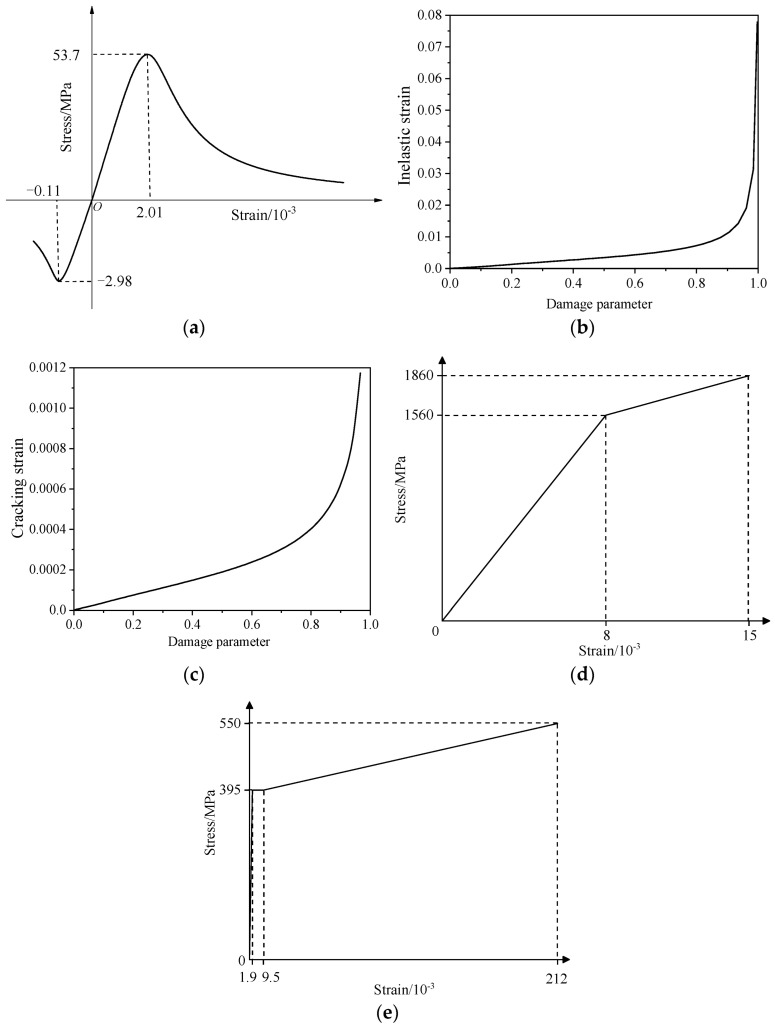
Constitutive curves of the finite element model. (**a**) Stress vs. strain of concrete; (**b**) Compression damage of concrete; (**c**) Tension damage of concrete; (**d**) Stress vs. strain of the pretensioned strands; (**e**) Stress vs. strain of steel bars.

**Figure 10:**
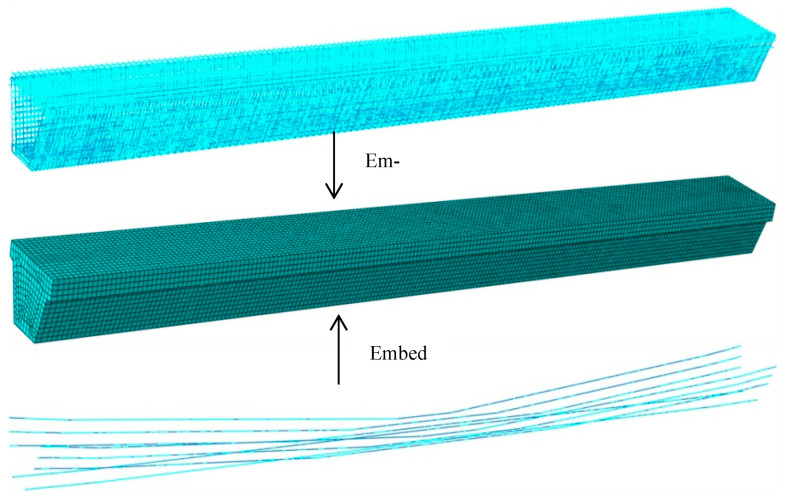
Refined model of the undamaged #3-2 girder.

To predict the change in the ultimate bearing capacity and mechanical properties of the repaired #3-2 girder strengthened by steel plates during the destructive process, and to compare the difference between the undamaged #3-2 girder and the damaged #3-2 girder, a model of the #3-2 girder in undamaged, damaged and repaired states was established by ABAQUS for a detailed analysis [35]. The modeling process of the undamaged #3-2 girder complied with the modeling process of the undamaged #4-1 girder. The modeling details of the undamaged #4-1 girder were described in Section 3.3 of another paper [24], and Figure 9 shows the stress-strain curves of the concrete, pretensioned strands and steel bars. Linear truss elements and eight-node hexahedral reduction integral elements were applied to establish the steel bars, pretensioned strands model and the concrete model, respectively. Considering the accuracy, convergence and computational efficiency of the numerical simulation, the mesh sizes of the concrete, steel bars and pretensioned strands were determined to be 120, 240 and 240mm, respectively, and the number of divided grids were 20,533, 18,065 and 810, respectively. The interface constraint of the steel bars, pretensioned strands and concrete are simulated by the embedding function. The effective stress of the pretensioned strands considering the prestress loss is shown in Table 3. For the box girder parts that were built, the pretensioned strands were subjected to a drop-temperature load to cause them to shrink, and the adjacent boundary was naturally strained to resist contraction, which realized the application of a prestressed load. The boundary condition applied to the girder end was a simply supported system; one end was a fixed hinge support, and the other end was a unidirectional sliding hinge support. Static-General in ABAQUS was used as a computational solver in analysis. The detailed finite element model of the undamaged #3-2 girder is shown in Figure 10.

### 4.2. Establishment of the Refined Model of the Repaired #3-2 Girder Strengthened by Steel Plates

**Figure 11:**
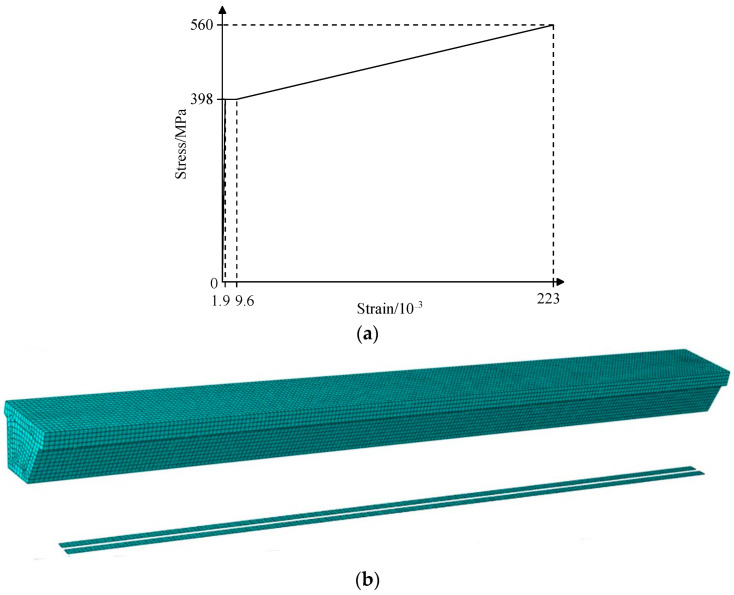
Refined model of the repaired #3-2 girder strengthened by steel plates. (**a**) Stress vs. strain curve of steel plates; (**b**) Refined model.

The process of modeling the repaired #3-2 girder strengthened by steel plates was basically the same as that of modeling the undamaged girder. The difference was in the simulation of broken pretensioned strands and strengthened steel plates. The broken pretensioned strands in the strengthened box girder were divided into two parts. Some of the strands were well bonded to the concrete, and they were regarded as the longitudinal steel bars. The temperature field was not applied, and the embedding function was used to constrain the strands with the concrete. The other strands separated from the concrete, and they were modeled using the life and death unit method. That is, the Model Change function in the software was used to passivate the prestress as it was applied. A four-node curved shell element was used to establish the strengthened steel plate model, and the stress–strain curves of the steel plates are shown in Figure 11a. According to relevant strengthening specifications [34], before the strengthened girder reached the ultimate bearing capacity, bond stripping failure between the strengthened steel plates and concrete was not allowed to occur, and the bond strength and shear strength of the adhesive were greater than the tensile strength and shear strength of the concrete. Therefore, the tie constraint was directly used to connect the reinforced steel plates and concrete, and the failure of the concrete was used to simulate the bond stripping failure between the strengthened steel plates and concrete. Figure 11 shows the finite element model of the repaired #3-2 girder strengthened by steel plates.

### 4.3. Establishment of the Model of the Damaged #3-2 Girder

**Figure 12:**
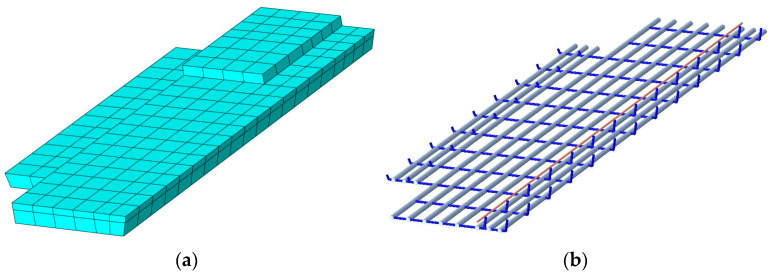

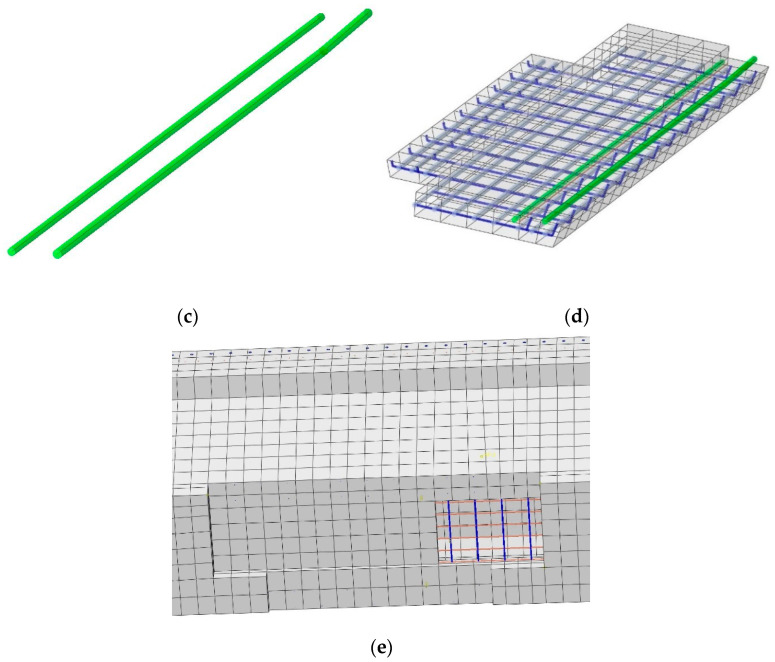
Details in the damaged district of the #3-2 girder. (**a**) The damaged part of concrete; (**b**) The damaged part of the steel bars; (**c**) The damaged part of the pretensioned strands; (**d**) The damaged part; (**e**) The damaged area of the girder.

The modeling of the damaged #3-2 girder was based on the model of the repaired #3-2 girder strengthened by steel plates. The main difference between them was that the simulation of the damaged #3-2 girder did not include reinforced steel plates, but the simulation included the spalling of damaged concrete and broken longitudinal steel bars. According to the actual condition of the damaged 3-2# girder, the damaged concrete spalled and the broken ordinary steel bars were cut, and the Model Change function of ABAQUS was used to model them. The model of the damaged #3-2 girder is shown in Figure 12.

### 4.4. The Results of Refined Finite Element Analysis

**Figure 13:**
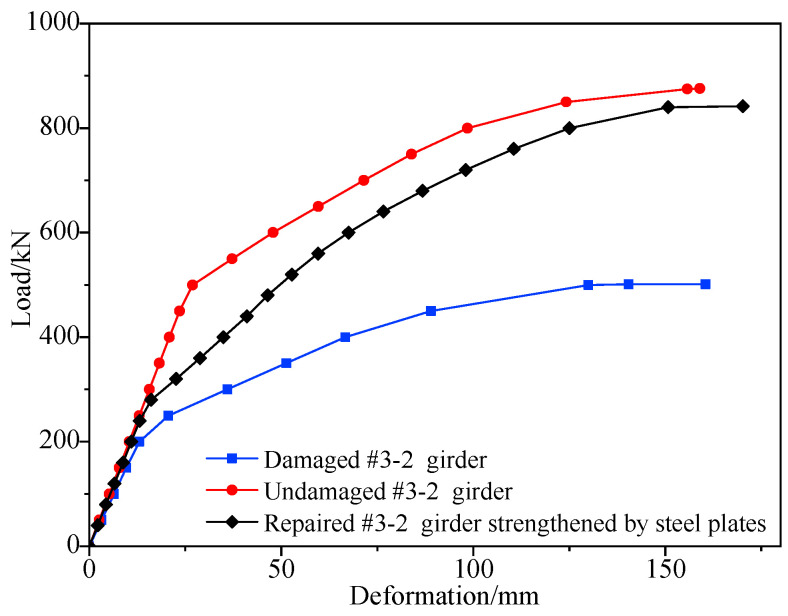
Deformation vs. load from the refined finite element analysis of the #3-2 girder in undamaged, damaged and repaired states.

Figure 13 shows the load vs. deformation curves of the repaired #3-2 girder strengthened by steel plates, the undamaged #3-2 girder and the damaged #3-2 girder were obtained in accordance with the nonlinear analysis. It shows that the destructive processes of the repaired #3-2 girder strengthened by steel plates and the damaged #3-2 girder were the same as that of the undamaged #3-2 girder, which included the elastic stage, the working stage with cracks and the destructive stage.

The deformation of the undamaged #3-2 girder was approximately 2.6 mm in each stage loaded with 50 kN, the deformation of the repaired #3-2 girder strengthened by steel plates was approximately 2.2 mm in each stage loaded with 40 kN and the deformation of the damaged #3-2 girder was approximately 3.3 mm in each stage loaded with 40 kN in the elastic stage. This indicated that the stiffness of the undamaged #3-2 girder in the elastic stage after being struck by a vehicle decreased by as much as 36%, but after being strengthened by steel plates, the girder that was injured had a 50% improvement in stiffness. The undamaged #3-2 girder’s cracking load was 361 kN, while the damaged #3-2 girder’s cracking load was 200 kN, or 44.6% less than the undamaged #3-2 girder’s cracking load. The cracking load of this repaired #3-2 girder strengthened by steel plates was 265 kN, which was 32.5% more than that of the damaged #3-2 girder, but there was still a large gap between the undamaged #3-2 girder, indicating that strengthened steel improved the cracking performance of the structure, but the improvement range was limited. When the load was increased to 875 kN, the concrete at the top of the undamaged #3-2 girder was crushed. The damaged #3-2 girder’s failure load was 552 kN, which was 36.9% less than that of the undamaged #3-2 girder. The failure load of the repaired #3-2 girder strengthened by steel plates was 840 kN, which was 52.2% more than that of the damaged #3-2 girder but 4% less than that of the undamaged #3-2 girder.

**Figure 14:**
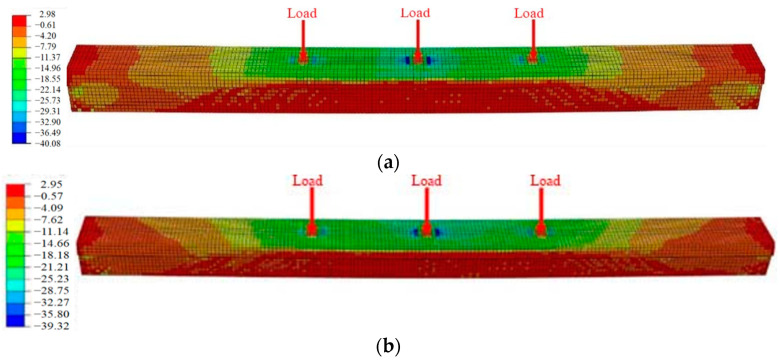

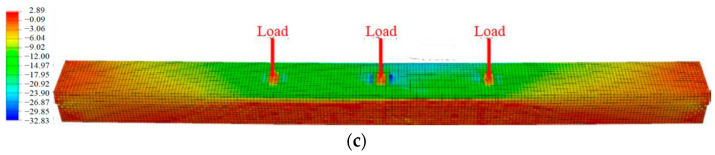
Stress diagrams of the concrete. (**a**) The undamaged #3-2 girder; (**b**) The repaired #3-2 girder strengthened by steel plates; (**c**) The damaged #3-2 girder.

**Figure 15:**
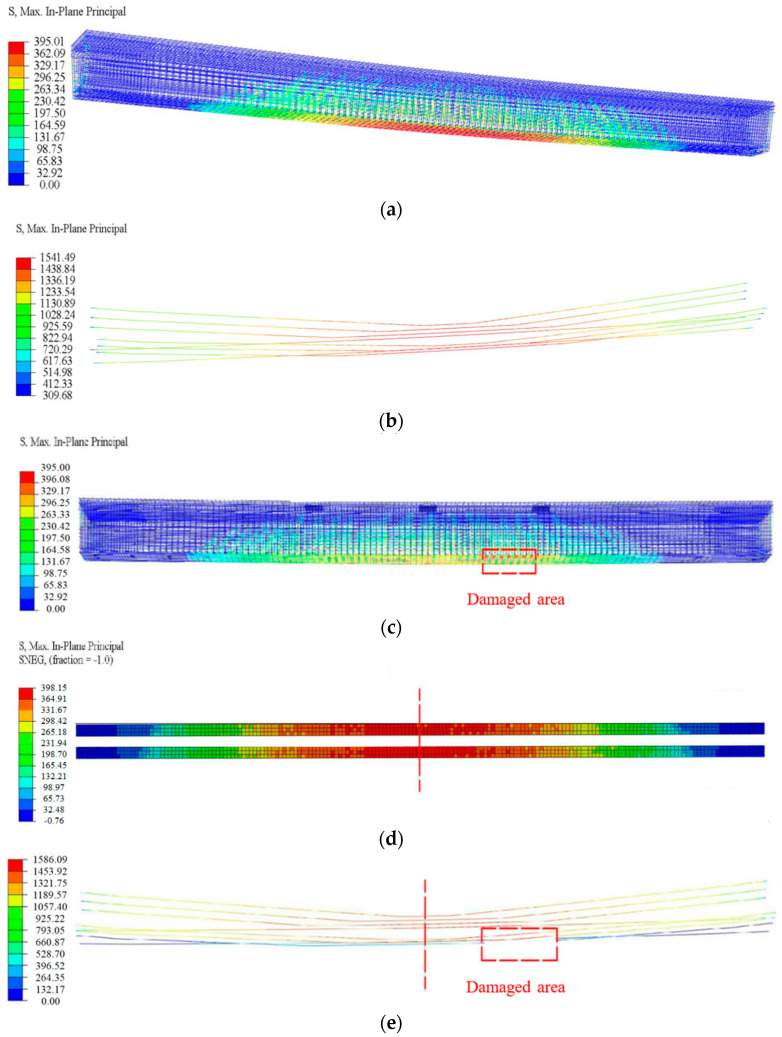

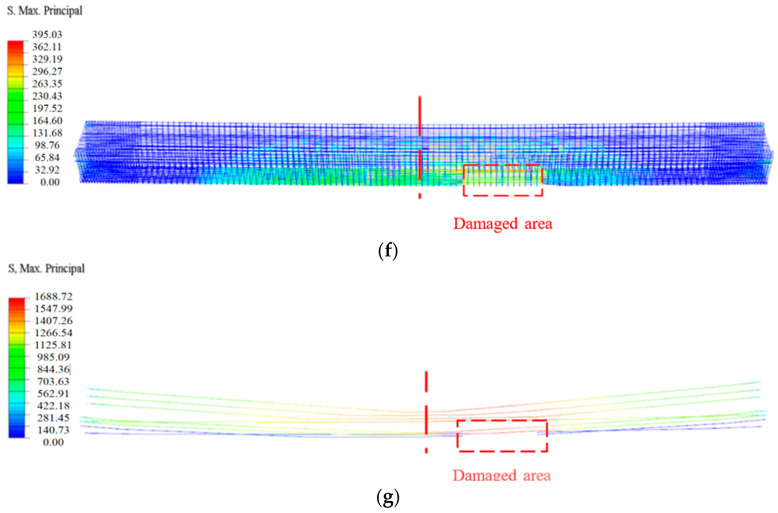
Stress diagrams of the steel bars, steel plates and pretensioned strands. (**a**) The steel bars of the undamaged #3-2 girder; (**b**) The pretensioned strands of the undamaged #3-2 girder; (**c**) The steel bars of the repaired #3-2 girder strengthened by steel plates; (**d**) The steel plates of the repaired #3-2 girder strengthened by steel plates; (**e**) The pretensioned strands of the repaired #3-2 girder strengthened by steel plates; (**f**) The steel bars of the damaged #3-2 girder; (**g**) The pretensioned strands of the damaged #3-2 girder.

According to the refined analysis, Figure 14 shows the concrete stress nephograms of the three states of the #3-2 girder when loaded to failure, and Figure 15 shows the stress nephograms of longitudinal steel bars, pretensioned strands and steel plates. Figure 14 shows that the maximum concrete compressive stress of the undamaged #3-2 girder reached 40.08 MPa, while that of the damaged #3-2 girder was 32.83 MPa, which was 18.08% less, and that of the repaired #3-2 girder strengthened by steel plates was 39.32 Mpa, which was 19.77% more than that of the damaged #3-2 girder. When the failure load was reached, the maximum tensile stress of the longitudinal steel bars of the three girders was 395 Mpa, which proved that the steel bars yielded. However, the pretensioned strands did not yield at this time. The tensile stress of the pretensioned strands of the damaged #3-2 girder was 1688.72 Mpa, while that of the repaired #3-2 girder strengthened by steel plates was 1586.09 MPa and that of the undamaged #3-2 girder was 1541.49 MPa. The results showed that the strengthening methods had a remarkable strengthening impact, effectively reducing the tensile stress of the pretensioned strands.

### 4.5. Ultimate Flexural Capacity

Table 4 shows the predicted values of the ultimate flexural capacity and cracking moment of the repaired #3-2 girder strengthened by steel plates, the undamaged #3-2 girder and the damaged #3-2 girder based on the refined analysis. It shows that the bearing capacity and the cracking moment decreased by 30.9% and 32.8%, respectively, for the damaged #3-2 girder compared with the undamaged #3-2 girder due to the collision by the overhigh vehicle. This indicates that structural damage such as concrete shedding, the fracture of steel bars and the fracture of pretensioned strands greatly reduced the flexural bearing capacity and crack resistance of the girder.

## 5. Destructive Test

### 5.1. Loading System

**Figure 16:**
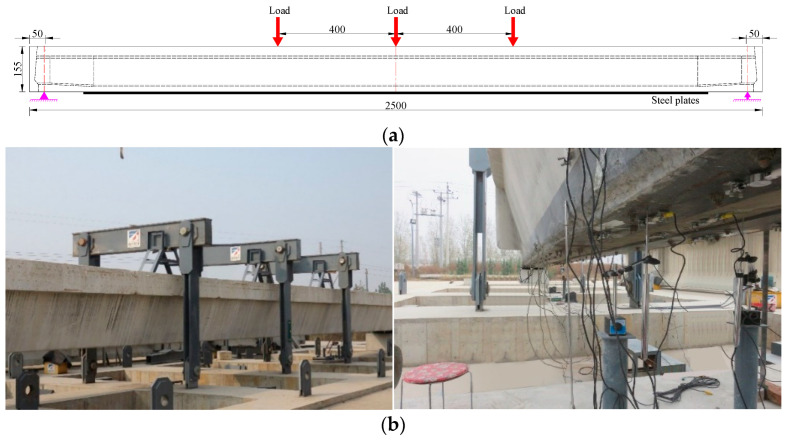
Loading layout of the destructive test. (**a**) Loading location (Unit: cm); (**b**) Field loading system.

To explore the recovery degree of the flexural capacity and the failure process of the repaired girder strengthened by steel plates, the repaired #3-2 girder strengthened by steel plates and the undamaged #4-1 girder were selected for a destructive test. Limited by the test site and conditions, three loading points were set for the destructive test of the repaired #3-2 girder strengthened by steel plates and the undamaged #4-1 girder, which were loaded by three jacks with a peak output force of 2000 kN. The distance between adjacent loading points was 4.0 m, and Figure 16a shows the loading position of the test box girder. The loading frame was assembled with slot steel and anchored on the concrete foundation, and its design bearing capacity was greater than the design load in the test, which ensured the safety and accuracy of the test. To prevent damage to the concrete near the loading position prior to the test from stress concentration, a steel plate with the following measurements was placed between the jacks and the girder: 60 cm × 60 cm × 3 cm. A pressure sensor with a range of 0~2000 kN was installed between the jack and the girder on the reaction frame. The test load and the test loading rate were controlled through measurements made by the pressure sensor during the test. The loading system is shown in Figure 16.

### 5.2. Loading Scheme

The loading procedure used the hierarchical loading method and was divided into three loading stages. First, the girder was loaded to 70% of the predicted value of the cracking load of the undamaged #4-1 girder and then unloaded. The second load was applied to 85% of the predicted value of the ultimate load and then unloaded, and the third load was loaded until the girder was damaged. During the loading of the undamaged #4-1 girder, 50 kN was applied at each stage. Considering that the repaired #3-2 girder strengthened by steel plates was repaired and strengthened based on the damaged girder, the load step was adjusted from 50 kN to 40 kN to ensure the safe and smooth progress of the test. When the load of each jack exceeded 450 kN, displacement control was employed to deliver the load until the box girder was damaged.

**Figure 17:**
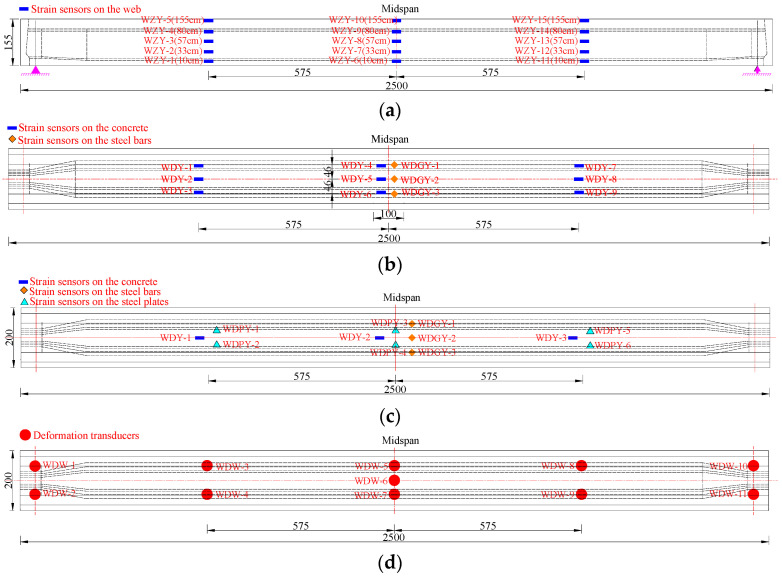
Sensor arrangements for the destructive test (Unit: cm). (**a**) Strain sensor arrangement at the concrete web; (**b**) Strain sensor arrangement at the concrete bottom and steel bars of the undamaged #4-1 girder; (**c**) Strain sensor arrangement at the concrete bottom and steel bars of the repaired #3-2 girder strengthened by steel plates; (**d**) Deformation sensor arrangement at the bottom of the girder.

The strain and deformation measurement points were arranged at the key section of the repaired #3-2 girder strengthened by steel plates and the undamaged #4-1 girder. To facilitate comparison and analysis with the load test results of the undamaged #4-1 girder, the web strain measurement points, bottom displacement measurement points and bottom steel bar strain measurement points of the repaired #3-2 girder strengthened by steel plates were consistent with those of the undamaged #4-1 girder. The concrete strain measurement points on the bottom plate were slightly adjusted. Figure 17 shows the precise locations and labels of the strain and deformation measurement points. The protective concrete layer at the installation area was removed before installing the steel bar strain sensors, and the steel bars’ surfaces were then sanded until they were smooth. Throughout the destructive test, all kinds of sensor data were monitored and collected.

### 5.3. Test Results

**Figure 18:**
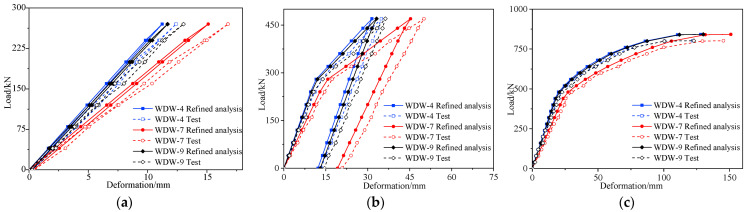
Deformation vs. load of the repaired #3-2 girder strengthened by steel plates. (**a**) Elastic stage; (**b**) Working stage with cracks; (**c**) Failure stage.

**Figure 19:**
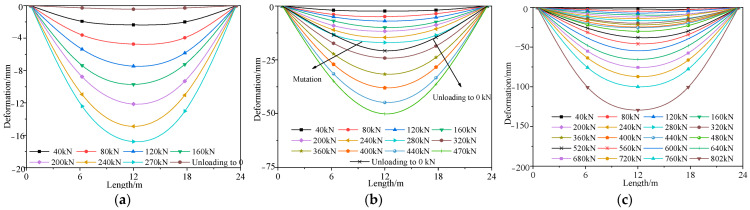
Deformation curve of the bottom plate of the repaired #3-2 girder strengthened by steel plates. (**a**) Elastic stage; (**b**) Working stage with cracks; (**c**) Failure stage.

Section 3,4 of a previous paper provided details of the destructive tests of the undamaged #4-1 girder [24]. Figure 18 shows the load vs. deformation curves of the midspan section of the repaired #3-2 girder strengthened by steel plates under each cyclic loading, which were obtained in accordance with the destructive test and compared with the results of the refined analysis, and Figure 19 shows the deformation curves along the girder length at each load stage. Figure 18 indicates that the destructive process of the repaired #4-1 girder strengthened by steel plates, which included the elastic stage, the working stage with cracks and the destructive stage when the steel bars succumbed, was exactly the same as the undamaged #3-2 girder. Moreover, the finite element analysis results for the girders after improvement were similar to the destructive test results. When each stage was loaded with 50 kN, the undamaged #4-1 girder deformed by around 2.9 mm in the elastic stage. When loaded with 40 kN, the repaired #3-2 girder that had been strengthened by steel plates deformed by approximately 2.5 mm in each stage. Cracks appeared at the bottom of the undamaged #4-1 girder when the load was 362 kN, and the cracking load of the repaired #3-2 girder strengthened by steel plates was 240 kN, which was 33.7% less than that of the undamaged #4-1 girder. The steel bars of the undamaged #4-1 girder began to yield when the load was 801 kN, and the steel bars’ yield load in the repaired #3-2 girder strengthened by steel plates was 740 kN, which was 7.6% less than that of the undamaged #4-1 girder. When the load reached 850 kN, the top concrete of the undamaged #4-1 girder was scrunched, and the failure load of the repaired #3-2 girder strengthened by steel plates was 802 kN, which was 5.6% less than that of the undamaged #4-1 girder. When the repaired #3-2 girder strengthened by steel plates was damaged, the maximum crack width was 1.53 mm, which was 24.6% less than that of the undamaged #4-1 girder, and the maximum deformation was 145.8 mm, which was 2.7% more than that of the undamaged #4-1 girder. Obviously, the girder’s ductility has improved, and the cracking degree was reduced by strengthening using steel plates. Figure 14 indicates that under symmetric load conditions, the deformation of the two girders maintained good symmetry.

Figure 18 also shows how the deformation of the undamaged #4-1 girder during the elastic stage in the refined model was approximately 2.6 mm in each stage loaded with 50 kN, which was 10.3% less than the destructive test result. The deformation of the repaired #3-2 girder strengthened by steel plates was approximately 2.2 mm in each stage loaded with 40 kN, which was 12% less than the result of the destructive test. The cracking load of the undamaged #4-1 girder was 361 kN, and that of the repaired #3-2 girder strengthened by steel plates was 260 kN. The failure loads of the undamaged #4-1 girder and the repaired #3-2 girder strengthened by steel plates were, respectively, 875 and 840 kN, which were 2.9% and 4.7% more than the destructive test results.

**Figure 20:**
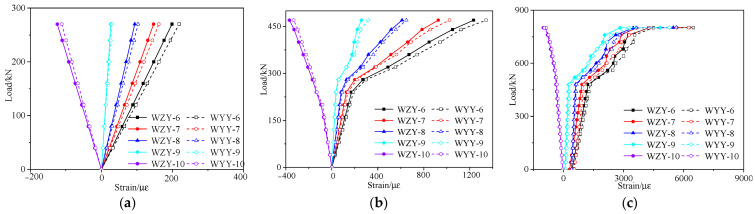
Strain vs. load curves of the concrete web at midspan of the repaired #3-2 girder strengthened by steel plates. (**a**) Elastic stage; (**b**) Working stage with cracks; (**c**) The failure stage.

**Figure 21:**
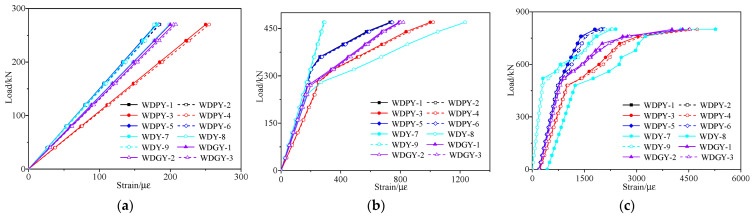
Strain vs. load curves of the repaired #3-2 girder strengthened by steel plates. (**a**) Elastic stage; (**b**) Working stage with cracks; (**c**) The failure stage.

Figure 20 and Figure 21 show, respectively, the strain vs. load curves for the web and bottom plate of the midspan section of the repaired #3-2 girder strengthened by steel plates that were obtained during the destructive test. Figure 20 indicates that the concrete strains of the right web under the same load were somewhat larger for the undamaged #4-1 girder and the repaired #3-2 girder strengthened by steel plates because the left web is taller than the right web. Figure 20 and Figure 21 show that the strains of the two girders exhibited an obvious three-stage development process. The strain vs. load curves of the undamaged #4-1 girder and the repaired #3-2 girder strengthened by steel plates, respectively, each showed an inflection point because of the appearance of cracks when the loads reached 362 and 240 kN. Before this inflection point, both the steel bars and concrete were in the elastic stage, and the strains and load states were basically linear. The reason that the crack load of the repaired #3-2 girder strengthened by steel plates was 33.7% less than that of the undamaged #4-1 girder was because of the fracture of the pretensioned strands. The steel bars of the undamaged #4-1 girder began to yield when the load reached 801 kN during the destructive test, and the steel bars’ strain was 2008 με. The yield load of the steel bars of the repaired #3-2 girder strengthened by steel plates was reduced by 7.6%, and the strain of the steel bars was 2009 με. In the serviceability state, the midspan strain of the repaired #3-2 girder strengthened by steel plates was 984.3 με, which was 29.6% more than that of the undamaged #4-1 girder.

### 5.4. Ultimate Flexural Capacity

The cracking moment and the actual ultimate flexural capacity of the box girders was calculated from the sum of bending moments by the combination of cracking load, actual failure load and dead load, and then compared with the theoretical value of the material’s design strength and the predicted value derived from the finite element analysis, as shown in Table 5. It shows that the values of the cracking moment and ultimate moment predicted by the refined analysis were similar to the destructive test results. The errors of the cracking moment and ultimate moment of the undamaged #4-1 girder were 3.4% and 2.5%, respectively, and the errors of the repaired #3-2 girder strengthened by steel plates were 5.4% and 3.9%, respectively. These results showed that the refined analysis accurately predicted the actual failure process and ultimate flexural capacity of the undamaged #4-1 girder and the repaired #3-2 girder strengthened by steel plates. The actual bearing capacities of the undamaged #4-1 girder and the repaired #3-2 girder strengthened by steel plates were approximately 1.60 and 1.63 times the theoretical values calculated according to the design strength, respectively, which indicated that each of the two girders had a large safety reserve. Compared with the undamaged #4-1 girder, the ultimate flexural capacity of the repaired #3-2 girder strengthened by steel plates reached 95.3%, the maximum deformation was 103% when the girder was damaged and the cracking load was only 66.3%, which indicated that the structural repair and strengthening measures basically restored the ultimate bearing capacity of the damaged girder to the level of the undamaged girder and improved the structural ductility. However, the cracking load of the damaged box girder did not improve to the desired level. The maximum bending moment of the undamaged single girder of the bridge under the combination of the six-axle vehicle load and the dead load was 6 084 kN·m, which was less than the design value of the bearing capacity of the component and met the specification. Additionally, the bearing capacities of the undamaged #4-1 girder and the repaired #3-2 girder strengthened by steel plates from the destructive test were approximately 2.2 and 2.1 times the maximum load effect, respectively.

## 6. Comparative Analysis

**Figure 22:**
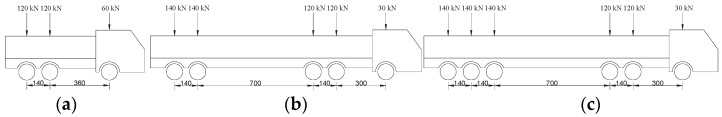

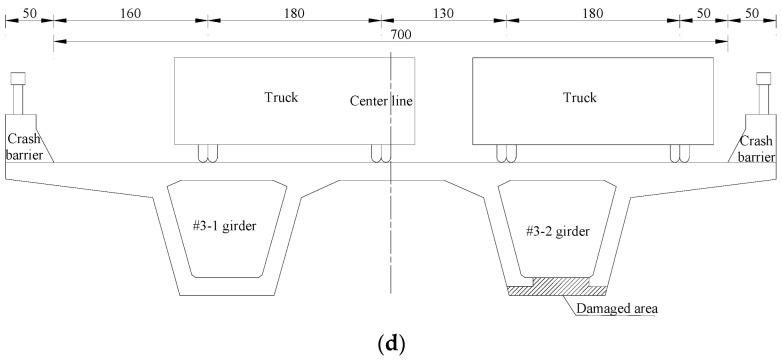
Different types of vehicles and horizontal arrangement. (**a**) Type 1; (**b**) Type 2; (**c**) Type 3; (**d**) Horizontal arrangement.

To further research the effect of the strengthening measures by steel plates for the improvement of the bearing capacity of the damaged girder and the degree of the recovery of the bearing capacity to the level of the undamaged #4-1 girder, the girders were compared and analyzed in terms of their flexural stiffness, flexural deformation and stress and crack width in accordance with the load combination given in the specification. Because the destructive test was performed after the girder had been strengthened with steel plates, the destructive test of the damaged #3-2 girder was lacking. However, according to research on the damaged #4-2 girder [24], which found that the refined analysis could simulate the actual damage process and bearing capacity of the damaged girder accurately, the results of the refined analysis of the damaged #3-2 girder were used instead of the results of the destructive test. The load included the dead load, secondary load, design live load and loads of three-axle, five-axle and six-axle vehicles given in the specification, which were denoted DL, SL, DLL, Type 1, Type 2 and Type 3, respectively. Schematic diagrams and lateral layouts of the vehicles are shown in Figure 22.

### 6.1. Stiffness

**Figure 23:**
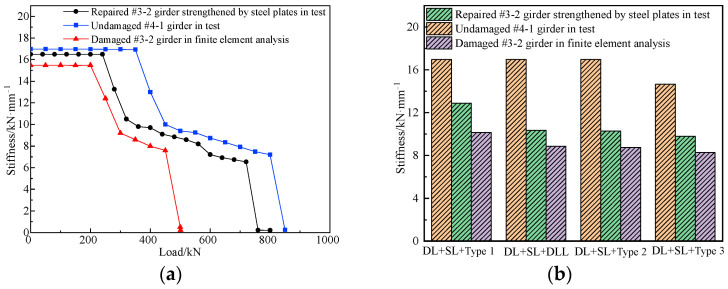
Stiffness comparison. (**a**) Load vs. stiffness curve; (**b**) Stiffness degradation under different live loads.

The flexural stiffness of bridges is an essential index used to assess the ability of bridges to resist deformation. Bridge damage is often accompanied by stiffness degradation, which can indirectly reflect the damage in the process of the bridge destructive test. According to the destructive test of the repaired #3-2 girder strengthened by steel plates, the undamaged #4-1 girder and the refined analysis of the damaged #3-2 girder, Figure 23 shows the load vs. stiffness curves for the three girders. It indicates that in the elastic stage, the flexural stiffness of the girders was the same as the initial flexural stiffness. Because of the repair of the broken steel bars and damaged concrete and strengthening by steel plates, the stiffness of the repaired #3-2 girder strengthened by steel plates increased by 6.6% compared with the damaged #3-2 girder, and reached 94.3% of that of the undamaged #4-1 girder. When the undamaged #4-1 girder, the repaired #3-2 girder strengthened by steel plates and the damaged #3-2 girder were loaded to 362, 240 and 200 kN, respectively, the flexural stiffness of the box girders decreased significantly, indicating that new cracks formed in the box girders, and the girders arrived at the working stage with cracks. The stiffness of the three girders showed a decreasing trend due to the continuous development of concrete cracking in the whole working stage with cracks. However, unlike the slow decline of the undamaged #4-1 girder and the damaged #3-2 girder, the stiffness dropped sharply due to the yielding of the steel plates when the repaired #3-2 girder strengthened by steel plates was loaded to 540 kN. When the undamaged #4-1 girder, the repaired #3-2 girder strengthened by steel plates and the damaged #3-2 girder were respectively loaded to 801, 740 and 410 kN, the flexural stiffness decreased sharply due to the yielding of the steel bars. Under the combination of different live loads, secondary load and dead load, the stiffness of the repaired #3-2 girder strengthened by steel plates increased by 27.1%, 17.5% and 18.3% of the stiffness of the damaged #3-2 girder and reached 72.7%, 51.9% and 56.4% of the stiffness of the undamaged #4-1 girder.

### 6.2. Flexural Deformation

**Figure 24:**
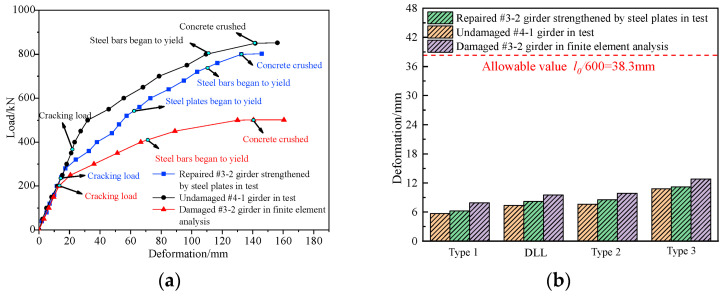
Midspan deformation comparison. (**a**) Load vs. deformation curve; (**b**) Midspan deformation under different live loads.

Basis on the refined analysis of the damaged #3-2 girder and the destructive test of the undamaged #4-1 girder and the repaired #3-2 girder strengthened by steel plates, Figure 24 shows the load vs. midspan deformation curves of the girders. For 0.7 times the 5-axle vehicle load, the midspan deformations of the undamaged #4-1 girder, the repaired #3-2 girder strengthened by steel plates and the damaged #3-2 girder were 7.6, 8.5 and 9.9 mm, and the flexure to span ratios were 1/3158, 1/2824 and 1/2424, respectively. The deformation of the repaired #3-2 girder strengthened by steel plates was 14.1% less compared with that of the damaged #3-2 girder and 11.8% more than the undamaged #4-1 girder, but both met the 1/600 requirement of the specification. For the 0.7 times six-axle vehicle load, the midspan deformations of the three girders were 10.8, 11.2 and 12.8 mm, and the flexure-to-span ratios were 1/2222, 1/2143 and 1/1875, respectively. The deformation of the repaired #3-2 girder strengthened by steel plates was 12.5% less compared with that of the damaged #3-2 girder and 3.7% more compared with that of the undamaged #4-1 girder. Thus, the deformations of the repaired #3-2 girder strengthened by steel plates for different live loads were significantly less than those of the damaged #3-2 girder, but slightly more than those of the undamaged #4-1 girder. The former difference was because the strengthening measures by the steel plates effectively increased the stiffness of the damaged #3-2 girder. The latter difference was because the broken pretensioned strands of the repaired #3-2 girder strengthened by steel plates were not repaired, which decreased the stiffness. However, the safety reserve of the repaired #3-2 girder strengthened by steel plates was still large.

### 6.3. Stress

**Figure 25:**
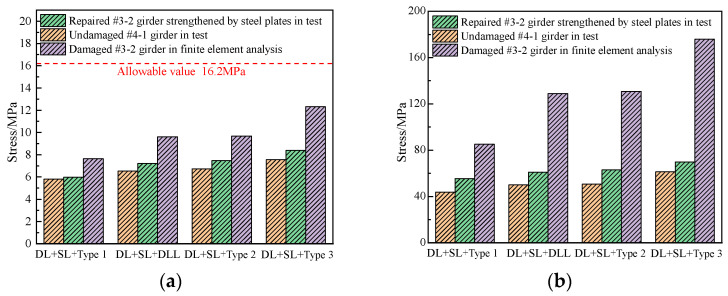
Stress comparison (**a**) Stress of concrete; (**b**) Stress of longitudinal steel bars.

Basis on the refined analysis of the damaged #3-2 girder and the destructive test of the undamaged #4-1 girder and the repaired #3-2 girder, Figure 25 shows the stress diagrams of the girders. It indicates that for the combination of 0.7 times the six-axle vehicle load, secondary load and dead load, the maximum concrete stress of the repaired #3-2 girder strengthened by steel plates was approximately 8.4 MPa, which was 31.9% less than that of the damaged #3-2 girder and 11.1% more than that of the undamaged #4-1 girder, however, all were far less than the allowable stress of 16.2 MPa in the design specification. The maximum steel bar stress of the repaired #3-2 girder strengthened by steel plates was approximately 69.8 MPa, which was 60.3% less than that of the damaged #3-2 girder and 13.8% more than that of the undamaged #4-1 girder. Thus, the steel plates were an effective strengthening measure. The reason that the stresses of the repaired #3-2 girder strengthened by steel plates were reduced compared to those of the damaged #3-2 girder was that the areas of the concrete and steel bars increased, which caused the stiffness of the whole section to increase, and the repaired concrete and steel bars partially bore the stress. The reason that the stresses of the repaired #3-2 girder strengthened by steel plates increased compared to those of the undamaged #4-1 girder was that the area of the strands decreased, which led to the reduction of the stiffness of whole section, and the remaining steel bars, strands and concrete bore greater stress.

### 6.4. Crack Width

**Figure 26:**
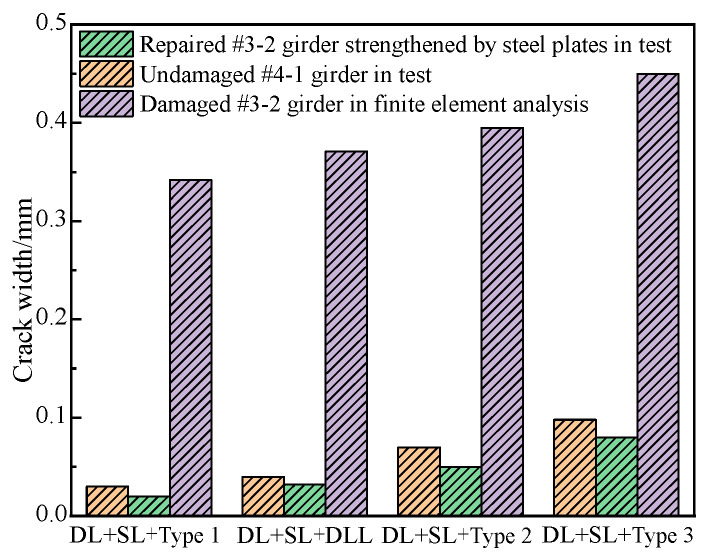
Crack width comparison.

Basis on the refined analysis of the damaged #3-2 girder and the destructive test of the undamaged #4-1 girder and the repaired #3-2 girder, Figure 26 shows the load vs. crack width diagrams of the girders. The maximum crack width of the repaired #3-2 girder strengthened by steel plates were approximately 0.05 mm and 0.08 mm, which were 87.3% and 82.2% less than that of the damaged #3-2 girder and 28.6% and 20% less than that of the undamaged #4-1 girder. This indicated that the crack resistance of the repaired #3-2 girder strengthened by steel plates not only recovered to the level of the undamaged #4-1 girder, but also improved to some extent. This was because the steel plates attached to the girder effectively protected the concrete and limited the development of cracks.

### 6.5. Traffic Load Capacity

To verify the recovery degree of the bearing capacity of the repaired #3-2 girder strengthened by steel plates, three standard vehicle loads were considered, i.e., three-axle, five-axle and six-axle vehicles. Then, the maximum bending moment effect under the combined action of the vehicle load, secondary load and dead load was obtained and compared with the flexural capacity of the repaired #3-2 girder strengthened by steel plates, the damaged #3-2 girder and the undamaged #4-1 girder to calculate the safety factor of the vehicles, as shown in Table 6.

Table 6 shows that the flexural capacity of the repaired #3-2 girder strengthened by steel plates through the destructive test was 62.6% more than that obtained according to the specification [32] and 36.7% more than that of the damaged #3-2 girder, reaching 95.3% of that of the undamaged #4-1 girder. This was because the strengthening measures by steel plates made the concrete, steel bars and strengthened steel plates bear the same force. The stiffness of the girder improved. The theoretical flexural bearing capacity of the repaired #3-2 girder strengthened by steel plates reached 93.7% of the level of the undamaged #4-1 girder, and the safety coefficient of the six-axle vehicle was less than 2.0. However, the actual safety coefficients of the three-axle, five-axle and six-axle vehicles were greater than 3.0 according to the destructive test, which indicated that the girder had a good capacity for vehicles.

## 7. Conclusions

The damaged #3-2 girder of an in-service prestressed concrete bridge with continuous girders was obtained by demolishing, repairing and strengthening. A destructive test of the strengthened #3-2 girder and the undamaged #4-1 girder was performed. Refined f analysis was conducted on the repaired #3-2 girder strengthened by steel plates, the undamaged #4-1 girder and the damaged #3-2 girder. The ultimate bending capacities of the three girders were compared and analyzed, and the following conclusions were drawn.

(1) The ultimate flexural bending capacities of the repaired #3-2 girder strengthened by steel plates and the undamaged #4-1 girder were obtained from a destructive test, and that of the damaged #3-2 girder was obtained by a refined analysis. The actual flexural bearing capacity of the repaired #3-2 girder strengthened by steel plates was 1.63 times the theoretical bearing capacity, which was calculated according to the specification [25], and 36.7% more than that of the damaged #3-2 girder, reaching 95.3% of that of the undamaged #4-1 girder. Although the theoretical flexural capacity of the repaired #3-2 girder strengthened by steel plates failed to meet the traffic requirements for six-axle vehicles, the actual safety factor was greater than three, which indicated a good capacity for vehicle traffic.
(2) The analysis showed that compared with the damaged #3-2 girder, the cracking moment of the repaired #3-2 girder strengthened by steel plates was 12.5% less, the stiffness in the elastic stage was 6.6% more and the maximum deformation was 9.2% less. Compared with the undamaged #4-1 girder, the cracking moment of the repaired #3-2 girder strengthened by steel plates reached 66.3%, the maximum crack width decreased by 24.6% and the stiffness in the elastic stage reached 94.3%. When the repaired #3-2 girder strengthened by steel plates finally failed, the maximum deflection was 2.7% more than the undamaged #4-1 girder. This showed that repaired and strengthening the damaged #3-2 girder improved the ductility, reduced the degree of cracking and increased the durability. Additionally, these results showed that first repairing the damaged #3-2 girder and then strengthening it with steel plates was an effective maintenance and strengthening measure.
(3) The finite element numerical analysis method based on the material testing results of the bridge reflected the mechanical properties and destruction process of the repaired #3-2 girder strengthened by steel plates and the undamaged #4-1 girder. The load vs. deformation curves obtained by this method were basically consistent with those of the failure test. The bearing capacities of the repaired #3-2 girder strengthened by steel plates and the undamaged #4-1 girder were accurately determined, and the errors with respect to the actual bearing capacities were 2.5% and 3.9%, respectively.
(4) Through destructive tests and a refined analysis, some conclusions about the repaired #3-2 girder strengthened by steel plates, damaged #4-2 girder and undamaged #4-1 girder were drawn. However, the improvement of the bearing capacity of an undamaged girder after strengthening has not been explored. Therefore, the destructive test and comparative analysis of the undamaged girder strengthened by CFRP will be carried out in the future.

## Figures and Tables

**Table 1:** Parameters for calculating the bearing capacity of the #3-2 girder in its undamaged, damaged and repaired states.

#3-2 Girder	*ƒ*_cd_/MPa	*ƒ*_sd_/MPa	*ƒ^′^*_sd_/MPa	*ƒ*_pd_/MPa	*A*_s_/mm^2^	*A^′^*_s_/mm^2^	*A*_p_/mm^2^	*h*_0_/mm	*a*^′^_s_/mm	*x*/mm
Undamaged	22.4	280	280	1260	4181	2670	4726	1253	45	142.4
Damaged	22.4	195	280	1260	707	2670	3475	1178	45	84.1
Repaired	22.4	280	280	1260	4181	2670	3475	1211	45	107.2

**Table 2:** Performance characteristics of the strengthening materials.

Materials	Design Strength/MPa		Measured Strength/MPa	Elastic Modulus/N·mm^−2^	Density/N·mm^−3^
	Tensile	Shear	Tensile		
Q345 steel plates	275	160	385	2.1 × 10^5^	7.7 × 10^−5^
Q345 anchor	160	–	–	2.1 × 10^5^	7.7 × 10^−5^

**Table 3:** Prestress loss.

Number of Strands	$\sigma_{\mathrm{con}}$ /MPa	$\sigma_{l1}$ /MPa	$\sigma_{l2}$ /MPa	$\sigma_{l4}$ /MPa	$\sigma_{l5}$ /MPa	$\sigma_{l6}$ /MPa	$\sigma_{\mathrm{pe}}$ /MPa
N1	1395	56.71	120.18	14.33	24.92	65.36	1113.49
N2	1395	56.71	120.18	14.33	24.92	65.36	1113.49
N3	1395	56.71	120.18	14.33	24.92	65.36	1113.49
N4	1395	33.63	92.67	14.94	30.71	68.38	1154.67

where $\sigma_{\mathrm{con}}$ is the strands’ tension controlling stress, $\sigma_{l1}$ is the frictional losses, $\sigma_{l2}$ is the anchorage losses, $\sigma_{l4}$ is the prestress loss caused by the concrete’s elastic compression, $\sigma_{l5}$ is the prestress loss caused by the prestressed strands relaxing, $\sigma_{l6}$ is the time-dependent loss caused by the concrete’s creep and shrinkage and $\sigma_{\mathrm{pe}}$ is the strands’ actual stress upon anchoring.

**Table 4:** The predicted values of the ultimate bending capacity and cracking moment of the #3-2 girder.

Bending Moment	Undamaged #3-2 Girder	Damaged #3-2 Girder	Refined Model of the Repaired #3-2 Girder Strengthened by Steel Plates
	Value/kN·m	Value/kN·m	Value/kN·m
Cracking moment	7272	4887	5748
Ultimate bending moment	13,831	9551	13,367

**Table 5:** Comparison of the predicted and experimental results.

Girder	Bending Moment	Theoretical	Refined Analysis		Destructive Test	
		Value/kN·m	Value/kN·m	Error/%	Value/kN·m	Error/%
Undamaged #4-1 girder	Cracking moment	6984	7272	4.1	7034	0.7
	Ultimate bending moment	8442	13,831	63.8	13,500	60.0
Repaired #3-2 girder strengthened by steel plates	Cracking moment	5124	5682	10.9	5417	5.7
	Ultimate bending moment	7913	13,367	63	12,864	53.6

**Table 6:** Safety coefficient for different types of vehicles.

Type of Truck	Bending Moment		Safety Coefficient					
			Ultimate Bearing Capacity of the Undamaged #4-1 Girder		Ultimate Bearing Capacity of the Repaired #3-2 Girder Strengthened by Steel Plates		Ultimate Bearing Capacity of the Damaged #3-2 Girder	
	The Dead Load	The Different Types of Vehicles	Theoretical Prediction	Destructive Test	Theoretical Prediction	Destructive Test	Theoretical Prediction	Refined Model
	/kN·m	/kN·m	(8442 kN·m)	(13,500 kN·m)	(7913 kN·m)	(12,864 kN·m)	(5127 kN·m)	(9551 kN·m)
1	2 053	1 720	3.71	6.66	3.41	6.29	1.79	4.36
2	2 053	2 301	2.78	4.97	2.55	4.70	1.34	3.26
3	2 053	2 947	2.17	3.88	1.99	3.67	1.04	2.54

## Data Availability

No new data were created or analyzed in this study. Data sharing is not applicable to this article.
